# Understanding the identity of lived experience researchers and providers: a conceptual framework and systematic narrative review

**DOI:** 10.1186/s40900-023-00439-0

**Published:** 2023-04-24

**Authors:** Veenu Gupta, Catrin Eames, Laura Golding, Beth Greenhill, Robert Qi, Stephanie Allan, Alison Bryant, Peter Fisher

**Affiliations:** 1 University of Liverpool, Liverpool, UK; 2 Liverpool John Moores University, Liverpool, UK; 3 University of Glasgow, Glasgow, UK; 4 Manchester Metropolitan University, Manchester, UK

**Keywords:** Lived experience, Service user, Service provider, Identity, Mental health, Research, Involvement, Conceptual framework, Systematic review, Narrative review

## Abstract

**Background:**

Identity is how we understand ourselves and others through the roles or social groups we occupy. This review focuses on lived experience researchers and providers and the impact of these roles on identity. Lived experience researchers and providers use their lived experience of mental or physical disability either as experts by experience, researchers, peer workers, or mental health professionals with lived experience. They must navigate both professional and personal aspects to their roles which can be complex. Performing roles simultaneously embodying professional and lived experiences contribute towards a lack of clarity to identity. This is not adequately explained by the theoretical evidence base for identity.

**Main body:**

This systematic review and narrative synthesis aimed to provide a conceptual framework to understand how identity of lived experience researchers and providers is conceptualised. A search strategy was entered into EBSCO to access Academic search complete, CINAHL, MEDLINE, PsycINFO, Psych Articles, and Connected papers. Out of the 2049 yielded papers, thirteen qualitative papers were eligible and synthesised, resulting in a conceptual framework. Five themes explained identity positions: Professional, Service user, Integrated, Unintegrated and Liminal. The EMERGES framework, an original conception of this review, found themes of: Enablers and Empowerment, Motivation, Empathy of the self and others, Recovery model and medical model, Growth and transformation, Exclusion and Survivor roots contributed to lived experience researcher and provider identities.

**Conclusions:**

The EMERGES framework offers a novel way to understand the identities of lived experience researchers and providers, helping support effective team working in mental health, education, and research settings.

**Supplementary Information:**

The online version contains supplementary material available at 10.1186/s40900-023-00439-0.

## Abstract (summary)

Patients now commonly help to teach healthcare professionals from their own perspective of what it is like to experience health difficulties and healthcare services. Consequently, the needs of patients are being better recognised by healthcare providers. Patients are also involved in research. These types of patient involvement lead to improved research and care. Patients included in this type of work are frequently referred to as patient providers, service user researchers, peer workers, experts by experience or lived experience researchers and providers. This might mean they are no longer viewed as people who use services but as people who provide a service. This review helps us understand how those in these roles are affected and how they understand themselves. We found they were sometimes described as either patients or professionals. Sometimes they were described as both patient and professional at the same time. Other times they were described as somewhere between a patient or professional. There were other important ideas that affected how they viewed themselves because of these experiences. These included feelings of empowerment and motivation from doing this important teaching and clinical work. The roles gave them a better understanding of their own experiences. These roles led to a personal growth and sense of achievement. The experiences of exclusion, and of being a patient affected how patient providers think of themselves. This understanding can lead to patient providers being better understood and leads to better teaching and training of healthcare professionals.

**Supplementary Information:**

The online version contains supplementary material available at 10.1186/s40900-023-00439-0.

## Background

“My fractured self was pieced together in my pursuit of my newly formed service user identity… I once had only a tiny seed of hope, now this has blossomed giving me a new sense of identity, purpose and direction.” ~ Alison Bryant, Service user Advisor (2023)

There is a movement to integrate lived experience into professional domains with many mental health professionals now speaking out about their own mental health experiences [1–3]. Service user and carer involvement is a mandatory requirement for all Health Care Professions Council (HCPC) regulated healthcare training programmes in the UK, including, clinical psychology, and social work training [4]. It was introduced after a commissioned review by HCPC into the benefits, facilitators, and barriers to service user involvement from healthcare contexts [5]. The sector finds service user involvement is integral to the effective training of healthcare professionals according to the British Psychological Society [6].

The involvement of lived experience providers in expert-by-experience roles occurs in universities, through professional bodies such as the BPS’s Group of Trainers in Clinical Psychology (GTICP) involvement group and patient and carer representatives within the Royal College of Psychiatrists (RCPsych). The BPS’s Division of Clinical Psychology [7] also released guidance on valuing the lived experience of trainee psychologists and how to integrate it into their work. Whilst healthcare professions’ training and service improvement is informed through service user feedback akin to consumer and market led approaches [8], disability activism is more concerned with emancipatory outcomes, achieving greater citizen control and rights for disabled people and survivors, led by democratic models [9]. The emergence of survivor-led work has occurred largely through opposition to psychiatry and the medical model [10]. It is important to acknowledge that the consumerist model of service user and carer involvement in healthcare training has arguably been co-opted from the survivor movement [11].

There is increasingly more participatory involvement in research and policy development within healthcare. In the UK, the National Institute of Health research (NIHR) [12] provides guidance and mandates the process of involvement in research. Externally other research organisations in the UK such as McPin Foundation, National Survivor User Network (NSUN), Survivor Researcher Network (SRN), and Shaping Our Lives (SOL), to name a few, integrate and value lived experience. Due to the exponential growth in lived experience work, understanding how this work impacts people in these roles is essential.

There are several different roles where lived experience might be present. The focus of this review is determining the identities of experts by experience, lived experience researchers, peer workers and mental health professionals with lived experience. Integration of lived experience in healthcare educational settings is achieved through the expert-by-experience role, where those with lived experience act as ‘critical friends’ to the organisation [13]. Their involvement provides trainee healthcare professionals with insight into the challenges and experiences service users and carers have whilst navigating their mental or physical disability and of using services [6]. The expert-by-experience role contradicts the traditional role of the service user and positions them as experts and people to learn from, as opposed to contexts where they are perceived as passive recipients of care [2]. The expert-by-experience role highlights the visibility of their lived experience, with an expectation to draw on this in their roles. In a similar way, the lived experience of peer workers is evident through their label, where they work in a relational way with patients they support in clinical settings. As they work from a lived experience lens this has implications for whether they are perceived as staff members, peers, or patients [14].

Comparatively, the lived experience researcher role requires the researcher to draw on their lived experiences in the research they conduct and through which they interpret data, working in professional and academic contexts. Though due to the visibility of their lived experience, their credibility as researchers and the knowledge they produce may be doubted for example by epistemic injustice [15] where they are perceived through the stigmatised lens of a service user and their place in a hierarchy. In contrast, those in mental health professional roles can also have lived experience [3] but have the privilege to choose whether to disclose this or not. Additionally, their placement in a hierarchy may afford them further privileges in comparison to those in other lived experience roles. Although they are likely to experience stigma on occasions of disclosure from other mental health professionals, and from survivors of mental disabilities [1]. Therefore, these different lived experience roles may vary in different ways, including the visibility of their lived experiences and the extent to which this is expected, acknowledged, or stigmatised.

These examples illustrate the contradictory positionings of lived experience researchers and providers and how they can be complex, leading to poorly understood identity constructions. Hodge [16] identifies limits to patient and provider roles that are dichotomous and assimilated into experiential and professional knowledge bases exclusively. The identity of the lived experience researcher and provider do not clearly fit into these exclusive categories. These different lived experience roles may therefore give rise to novel formations of identity, requiring greater lucidity.

Benefits to integrating lived experience in mental health education and research results in empowerment [17], improved empathetic responses from healthcare professionals, influencing mental health institutions to be person-centred [6] and supports the learning of healthcare professionals [18]. Oliver et al. [19] describe the benefits of service user involvement but highlight the negative aspects of this work, including practical and personal risks to those engaged in this work. Resistance to the integration of lived experience by some service providers occurs through exclusion and tokenistic involvement [20], and queries over fitness to practice [14, 21–23]. In addition, sometimes there are queries regarding the representativeness and authenticity of service users who are considered too professionalised [24]. These roles can cause emotional burden to those that perform them [25].

Further barriers to meaningful integration of lived experience can occur for several reasons. Service providers may want to maintain positions of power, they may lack experience in this type of work, or involvement may be at odds with the models within which they work, such as the medical or recovery model. Service providers may lack funding for these roles or may have negative views on the benefits of lived experience, or even vary over their subjective conceptual understanding of what it means to integrate lived experience [26, 27].

These illustrations of risk to the lived experience researcher or provider, suggest the role may be unsafe and cause harm. Richards et al. [3] reported that the mental health sector is not yet ready or safe regarding integration of lived experiences. It is essential, and of great ethical necessity, that service providers do not cause harm to service users in these contexts. These roles, in which there is integration of lived experience in professional spaces, is likely to impact identity, an under researched area.

### The theoretical basis of identity

Research on identity of healthcare professionals has tended to focus on the development of professional identities in, for example, medical students [28, 29] nurses [30], clinical psychology trainees [31], and social workers [32]. This research identifies the importance of clarity regarding identity, resulting in better team working, wellbeing and resilience. Additionally, experiences of mental illness, such as Psychosis, also influence changes in identity [33]. Mental illness and disability are the basis for undertaking lived experience researcher and provider roles and so it is essential to understand how these roles might further influence identity.

Identity theories suggest identities are formed via group membership [34], or the roles we occupy [35], and intersectional [36] and liminal [37] processes. Social Identity theory [34] can be used to explain the service user identity, who may self-define with an expert by experience group as their in-group, and from which they begin to share similar values, beliefs, and behaviours. They identify differences between themselves and others. For example, experts by experience in clinical psychology may find themselves in opposition to psychiatry. Tse, Cheung, Kan, Ng and Yau [38] find service user involvement provides the right context to lead to changes in identity. Social Identity theory suggests identities are formed in opposition to other social identities. However, the theory does not account for simultaneously occupying the oppositional positions of lived experience and professional identities.

Identity theory [35] suggests identity is drawn through the roles we occupy in a structured society. Individuals attribute meaning and expectations to these roles through interactions with others. The expert by experience can be seen to move between different identities such as the patient and professional, dependent on context and whom they are talking to [20]. We seek to preserve the clarity of one’s own role, resulting in more certainty and satisfaction with our own identities [39]. The lived experience role, however, spans both patient and professional, resulting in contradictory meanings which are likely to be unsatisfactory, due to a lack of clarity. This suggests a complexity to these roles that identity theory may not completely account for.

Liminality [37] better accounts for ‘in between states.’ Liminality is understood as “a position of ambiguity and uncertainty” (Beech [40]; p. 287). The concept describes the role of peer workers and peer researchers [14, 25]. Liminality may be useful in explaining lived experience roles. Although, the concept undermines the complexity of the lived experience researcher or providers’ identity due to the suggestion of an absence of identity.

Intersectionality [36] refers to the intersecting personal elements of an individual such as race, gender, class, ethnicity, sexuality, and others, that in conjunction with each other, compound the experience of discrimination. Mental health identities and professional identities may also be influenced by intersectionality [41], suggesting the role may be burdensome. Additionally, Liminality [37], may also be burdensome, and interact and impact identity, similarly to the process of intersectionality [41].

The theories do not explain the identity of lived experience researchers and providers. Considering lived experience researcher and provider roles are increasingly common, a better understanding of how these roles impact identity is required. This will support others to better understand those in these roles, encourage better team working and identify influencing factors relating to the formation of identity.

Systematic reviews have focussed on service user involvement, the process of involvement and, but to a lesser extent, on the impact of involvement on learning and clinical skills [42–45]. A systematic review and conceptual framework of recovery of mental health patients has been conducted [46], a conceptual understanding of identity changes in psychosis [33], and a literature review into service user involvement and identity [47]. However, currently, there is limited research into the identity formation of lived experience researchers and providers and no systematic review that provides a synthesis and conceptual framework of factors relating to their identity development. This systematic narrative review will combine articles that focus on identity across different roles that features lived experience, including experts by experience, lived experience researchers, peer workers and mental health professionals with lived experience. It will explore the similarities and differences relating to their identity constructions. This will be a starting point from which to identify how identities across these groups are constructed and offer a contribution to the limited research in the area. This will further elucidate and give clarity to the identities of lived experience researchers and providers.

*Aims* The aim of the review is to identify how identity has been conceptualised in relation to lived experience researchers and providers in mental health, education, and research settings.

The secondary objectives are to develop a conceptual framework to describe the identities of lived experience researchers and providers.

## Main body

### Methodology

#### Conditions or domain being studied

The systematic review explored lived experience researchers and providers and how their identities were impacted by these roles in mental health, education, and research.

#### Positionality and reflexivity

The review was conducted by VG, PhD researcher, and PF, LG, BG, Clinical psychologists, and CE, research psychologist. VG is also an expert by experience for two involvement groups for clinical psychology programmes and is a service user advisor to national research projects related to their own individual experiences. The research team see this as a strength but are aware of the potential biases each of their own experiences may cause in relation to the design and analysis of the research. This was mitigated by themes and findings of this review being discussed as a team. The review held pragmatism as its epistemology to understand findings and collate studies that differed in methodologies and philosophical perspectives. SA and RQ were independent PhD students each with their own lived experience and academic knowledge contributing to quality appraisal alongside VG. Service user advisor AB also brought her own lived experience to help assess validity of the findings. The GRIPP2 checklist also details the nature of lived experience within the systematic review. VG also kept a reflective diary throughout the process to better understand the emerging themes and relationships between the data, as well as to record feedback from across the research team discussions and those with lived experience.

#### Information sources

**Fig. 1:**
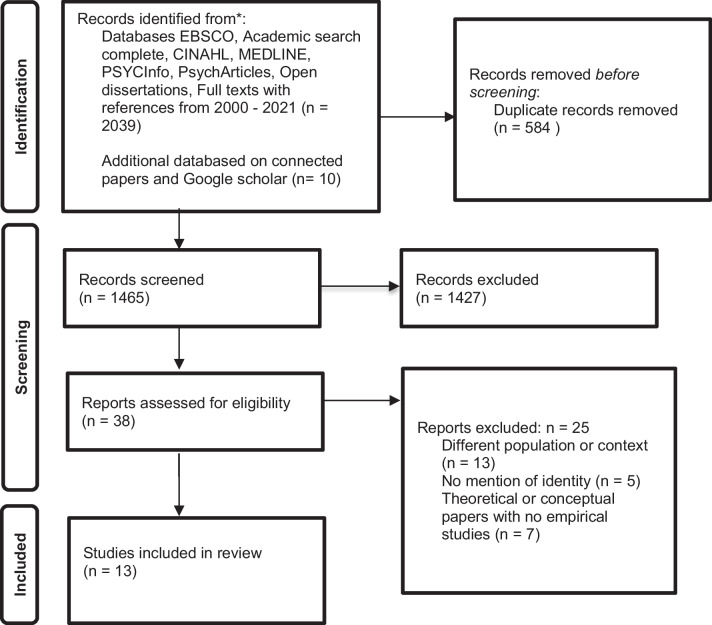
PRISMA Flow diagram of selection process

The search strategy was trialled and tested in an iterative way until it was optimal in capturing relevant articles for the review. The search strategy was co-created with the research team and a university librarian was also consulted. The search strategy is detailed in Table 1, which was inputted into the University of Liverpool database, on 21st November 2021 and re-run on 17th May 2022 using EBSCO to access journals from Academic search complete, CINAHL, MEDLINE, PsycINFO, Psych Articles, and University of Liverpool Catalogue was used for this. Hand searching of references from papers was conducted. Connected papers website was used to search for related papers. Figure 1 reports the selection procedure.

**Table 1:** Search strategy

1	Service user involvement OR Service user participation OR Service user engagement OR Service user advisors OR Expert by experience involvement OR Expert by experience participation OR Expert by experience engagement OR Expert by experience advisors OR Patient involvement OR Patient and Public Involvement OR Patient and Public participation OR Patient and Public engagement OR Patient and Public advisors OR Service user and Carer involvement OR Carer involvement OR Carer participation OR Carer advisors OR Coproduction OR Collaboration OR Peer worker OR Peer support OR Peer researchers OR Peer engagement OR Peer participation OR Peer involvement Or Survivor researcher OR Survivor participation OR Survivor involvement OR Survivor engagement
2	Clinical psycholog* OR Social work OR Mental health nurs* OR Research OR Service provider OR Mental health professional
3	Training OR Education OR Mental health education
4	Identity OR Mental health identity OR Service user Identity OR Recovery Identity OR Illness Identity OR Dual Identity OR Becoming OR Identity construct

This search strategy was put in place with and/or terms as follows: 1 and 2 and 3 and 4

#### Selection process

VG initially assessed eligibility of studies using criteria established in Table 2. Where there were queries over eligibility the supervisory team were consulted, PF, CE, LG, and BG.

**Table 2:** Eligibility and Inclusion and Exclusion criteria

	Inclusion criteria	Exclusion criteria
S	*Sample* Population of sample were lived experience researchers and providers (defined as those with mental or physical disability). Mental or physical disability are not specified. Participants aged 18 years old and above	
	Lived experience researcher and providers who may also be referred to as experts by experience, peer researchers, co-researchers, lived experience researchers, service user researchers, disability researchers, survivor researchers and/or practitioners with lived experience including mental health professionals with mental or physical disabilities and also peer support workers and peer workers. In the context of mental health, education and research	Lived experience researchers and providers in the context of physical healthcare conditions and contexts outside of mental health, education and research. Medical students and medical doctors
PI	*Phenomenon of Interest* The study had to explore the effect lived experience researcher and provider roles on identity	Studies that did not focus on the effect of lived experience roles on identity. Studies on identity in relation to aspects of identity such as LGBT, gender, social class and ethnicity, national and political. Studies that used Erikson’s model of identity development and Freud’s Id, Ego and Superego theory
D	*Design* Qualitative interviews, not limited by design, methodology or philosophical epistemology	
E	*Evaluation* Conceptualisation of identity, causes, effects, related factors, sample size, year published, methodologies, philosophical epistemology, key findings and study location Published in the English language but not limited by country of origin	
R	*Research type* Peer reviewed studies Qualitative empirical studies Published between January 2000 and May 2022	Purely theoretical or conceptual papers and the grey literature

SPIDER tool for qualitative research, Cook et al. [43]

#### Data items

The characteristics of the studies, type of study, method used, sample size, participant demographics, research aims, and findings were extracted. More specifically, effects and impact of lived experience researchers and providers’ work on identity were extracted through preliminary summaries and themes extracted of each study.

#### Synthesis methods

The review followed the PRISMA [48] protocol for conducting systematic reviews. The modified version of Popay et al. [49] stages of developing a conceptual framework was applied to the synthesis. The stages were (1) Develop a preliminary synthesis of findings, (2) Explore relationships in the data within and between studies and (3) Assess the robustness of the synthesis. This methodology was used as it is a systematic way to conduct a narrative synthesis and reports the process transparently. This methodology has also previously been used successfully in a systematic narrative review to develop a conceptual framework before [33]. Stages 1 and 2 were undertaken by VG.

### Stage 1 preliminary synthesis

This stage involved tabulation and a thematic analysis of the identity of lived experience researchers and providers. An overview of the characteristics and themes of each study are in Additional file 1: Table S1. This preliminary synthesis informed the development of a coding framework and each article within the review inductively coded to identify additional themes, using NVIVO, this allows for new and emerging themes and a flexible coding approach to the different articles.

### Stage 2 exploring relationships within the studies

The studies were assessed for similarities and differences to identify emerging themes that explain identity. The studies in the four different groups, mental health professionals with lived experience, peer workers, lived experience researchers, and experts by experience, were analysed separately in this sequential order. The results were compared and synthesised to see if the research areas held different or similar conceptualisations of identity, and these supported in translating the initial synthesis into a conceptual framework. Codes across the articles that were similar in meaning were brought together and labelled under overarching themes. The methodology followed allowed for flexibility, allowing the researchers to pre-define the methodology of the three stages, as detailed, which were rigorously followed.

### Stage 3 certainty assessment: checking the robustness of the synthesis

Quality appraisal was conducted using Joanna Briggs Institute (JBI) critical appraisal tool [50] by three independent researchers, VG, SA and RQ, using the same procedure and variation discussed until consensus was reached over the course of two meetings. The reviewers were each PhD researchers and two of these reviewers with lived experience. To understand the robustness of the synthesis service user advisors (1), lived experience researchers (3), and academic mental health professionals (3) were consulted to see whether the conceptual framework fitted with the way they understood their identities as lived experience researchers and providers. This involved gaining feedback and asking the question ‘Does this fit with how you understand your experiences as a lived experience researcher/provider?” The purpose of this was to check the validity of the conceptual framework. This is further evidenced through a reflective account by Service user Advisor, AB.

### Eligibility and inclusion and exclusion criteria

The SPIDER tool for qualitative research, Cooke et al. [51] was used to describe the eligibility of articles within the review. This is commonly used in qualitative syntheses. The review only included articles with a focus on identity of those who performed lived experience researcher and provider roles in the context of mental health, training, and research settings and not for example in medical settings. Only participants of adult age were included. This is detailed in Table 2.

## Results

The search strategy yielded 2049 articles from the databases and after duplicates were excluded resulted in a total of 1465 articles. Of the 1465 articles screened, thirteen articles met the inclusion criteria, as shown in Figure 1. These were published between 2011 and 2022 and originated from USA, Australia, UK, Canada, Finland, and Belgium, and sample size of the studies ranged from 1 to 46 participants. These studies explored the identities of mental health professionals who were also survivors or service users [1, 3]; the identities of peer workers [14, 21, 22] social work students [23] service user researchers, experts by experience and co-researchers and peer researchers [20, 25, 52–55] and service user and carer representatives [56]. All studies were qualitative but varied in methodology, epistemology, and analysis and so a pragmatic approach was used to synthesise different approaches. One of the studies was part of a randomised controlled trial and another part of a pilot study and all, empirical qualitative studies. A synthesis of the findings follows, followed by a translation of these findings into a conceptual framework.

### Quality appraisal

The Joanna Briggs Institute (JBI) [50] quality appraisal tool for qualitative research was used to assess the quality of each paper using 10 items that were scored as yes, unclear, and no regarding quality. Three independent researchers assessed the 13 papers. Following discussion over differences in ratings, consensus was reached. Fleiss’ Kappa interrater agreement was moderate, k = 0.485, *p* < 0.001. Kappa agreement for ratings of No, k = 0.769, *p* < 0.001, Yes, k = 0.554, *p* < 0.001 and Unclear, k = 0.184, *p* < 0.001. The mode quality appraisal ratings can be seen in Table 3. Ethical approval was not stated in some studies [1, 20, 23]. There was some bias in the recruitment process [1] for example this study recruited participants from their own personal networks. Each of the studies lacked diversity in their sample regarding ethnicity and gender. 8 studies did not report demographics on ethnicity [1, 20–23, 52–54]. 3 studies did not report gender [1, 23, 53] and 1 study reported this ambiguously [52] and Cooke et al. [55] included a sample of only white females. Age was not reported in 5 studies [1, 3, 21, 23, 25] and 1 study reported that participants were of adult age, but no descriptive statistics were included [22]. Each of the studies varied in philosophical approaches and methodologies. There were also limited statements identifying the researchers’ own positionality in relation to the research, either theoretically or culturally, [23, 25] and it was unclear in 3 studies [14, 22, 52] and very limited acknowledgement of the researcher’s impact on both the research and vice versa, which was not identified in 4 studies [14, 23, 25, 52]. Different lived experience researcher and provider roles were treated as a homogenous group within 2 studies [1, 3]. All but one study allowed for heterogeneity regarding type of lived experience of mental or physical disability. Cooke et al. [55] only included people with a personality disorder diagnosis. 2 studies were heavily theoretically driven without the researchers acknowledging the deductive approach they used and its influence on findings [14, 56].

**Table 3:** Mode responses to quality appraisal

JBI Quality appraisal checklist for qualitative research	Adame [1]	Newcomb et al. [23]	Richards et al. [3]	Simpson et al. [14]	Wilson et al. [22]	Toikko [52]	Jones et al. [53]	Hutchinson et al. [54]	Cameron et al. [20]	DeRuysscher et al. [21]	Faulkner & Thompson [25]	Hill et al. [56]	Cooke et al. [55]
1. Is there congruity between the stated philosophical perspective and the research methodology?	✓	?	✓	✓	✓	✓	✓	✓	✓	✓	✓	✓	✓
2. Is there congruity between the research methodology and the research question or objectives?	✓	✓	✓	✓	✓	✓	✓	✓	✓	✓	✓	✓	✓
3. Is there congruity between the research methodology and the methods used to collect data?	✓	✓	✓	✓	✓	✓	✓	✓	✓	✓	✓	✓	✓
4. Is there congruity between the research methodology and the representation and analysis of data?	✓	✓	✓	✓	✓	✓	✓	✓	✓	✓	✓	✓	✓
5. Is there congruity between the research methodology and the interpretation of results?	✓	✓	✓	✓	✓	✓	✓	✓	✓	✓	✓	✓	✓
6. Is there a statement locating the researcher culturally or theoretically?	✓	×	✓	?	?	?	✓	✓	✓	✓	×	✓	✓
7. Is the influence of the researcher on the research, and vice versa addressed?	✓	×	✓	×	✓	×	?	✓	✓	✓	×	✓	✓
8. Are participants and their voices adequately represented?	✓	✓	✓	✓	✓	✓	✓	✓	✓	✓	✓	✓	?
9. Is the research ethical and according to criteria for recent studies and is there evidence of ethical approval by an appropriate body?	×	×	✓	✓	✓	?	✓	✓	?	✓	✓	✓	✓
10. Do the conclusions drawn in the research flow from the analysis or interpretation of the data?	✓	✓	✓	✓	✓	✓	✓	✓	✓	✓	✓	✓	✓

### Stage 1 Preliminary synthesis

Stage 1 involved summarising, tabulating, and data extraction of the studies in the review according to the pre-defined methodology. This is presented in Additional file 1: Table S1.

### Stage 2: Exploring relationships within the studies

Stage 2 involved synthesising these studies and identifying similarities and differences, in the relationships within and between the studies. Five main themes of identity positions were identified and seven themes relating to identity development. The following section explores these themes across the studies. Table 4 details the positions of identity found, and Table 5 identifies how the studies contribute to the development of the EMERGES framework.

**Table 4:** A Translation of findings into the positions of identity

Positions of identity and causal factors	Studies and their original conceptions
Professional	Newcomb et al. [23] Positive/Negative role modelling; Richards et al. [3]; Simpson [14] Occupational training. Wilson [22] Peer worker. Toikko [52] Combining experiences with existing competences. Jones [53] Competences and skills. Use of existing skills in involvement work [55, 56]
Service user	Richards et al. [3] Patient. Wilson [22] Drug user. Cameron et al. [20] disability identity locates problem in individual, activists reframe this to society not meeting their needs and being the reason for impairment. Cooke et al. [55]
Integrated	Richards et al. [3] Personhood; Adame [1] Benefits of disclosure: Newcomb et al. [23] Personal experiences help professional identity. Embodied experiences [25]
Unintegrated	Richards et al. [3] Adame [1] Risks of disclosure; Newcomb et al. [23] Disclosure difficulties, Not easily integrated. Reluctance to share lived experience [55] Toikko [52] Creating distance from experience. Alienation [25]
Liminality	Simpson [14] Liminality of PSWs. Transgressive and ambivalent Faulkner andThompson [25]

**Table 5:** A Translation of findings into the EMERGES framework

The EMERGES Framework as causal factors	Studies and their original conceptions
Empowerment	Simpson [14] Occupational training leads to competence, empowerment, skills, knowledge. Toikko [52] Developing an orientation to the future and politicised identities. Jones [53] politicised identities through shared grievances and collective identity. Hutchinson and Lovell [54] Reframing of illness, sharing experiences and listening to each other’s stories enabled and empowered co-researchers to be less critical of the self and normalise experiences of distress. Cameron et al. [20] Good outcomes of involvement lead to empowerment, purpose, value, skills, and knowledge. DeRuysscher et al. [21] Life rebuilding. Disempowering through the system and bureaucracy but enablers through personal, collective, work, and system level strategies [25] Hill et al. [56] feeling listened to, valued and with purpose and making a difference. Cooke et al. [55] impact on self and others
Motivation	Newcomb et al. [23], Simpson [14] Model recovery and inspire others. Toikko [52] Motivation to share experience and reduce stigma/raise awareness. Jones [53] Motivation to move from illness identity to a positive one. Cooke et al. [55] Taking up the trainer role—It just all took off
Empathy of the self and others	Simpson [14] (Identity and relationships, connection with peers); Wilson [22] Drug talk can be triggering. Toikko [52] Sharing experiences with peers and friends. Jones [53] Sharing of experiences leads to common shared experiences and politicised identities. Hutchinson and Lovell [54] Unrestricting lives and Reciprocity-connections with others affirmative experiences and belief in others and the self, hearing others’ stories enabled empathic connections and normalised experiences of distress. Cameron et al. [20] find that social connections are a result of involvement. Hill et al. [56] being understood by trainees and feeling connected to each other as survivors. “Band of brothers” [55]
Recovery model/Medical model	Adame [1] Differences between psychological and psychiatric models; Richards et al. [3] Jones [53] Cameron et al. [20] service providers do harm when reverting to the medical model lens and resulting in diminished identities. Decisions, diagnoses being made for them in secrecy [55]
Growth and Transformation	Richards et al. [3] Personhood; Jones [53] Becoming an EBE changed illness identity to a more positive one. Hutchinson and Lovell [54] process of hearing others’ stories humanised the experience of distress and transformed and reframed service user identities. Hill et al. [56] I am not the same person I was. Emergence of professional identity linked to value and power [55]
Exclusion/Stigma and Discrimination	Richards et al. [3] Unintegrated; Adame [1] Us and Them divisions; Newcomb et al. [23] Disclosure difficulties. Simpson [14] Identity and relationships (PSWs Excluded by other professionals). Wilson [22] Barriers to accessing services when relapsing as a PSW, Difficult to move beyond Drug user identity to professional opportunities. Jones [53], Cameron et al. [20] service providers choose who is listened to and who has power. Alienation and exclusion of diversity in white spaces [25]. Hill et al. [56] breaking the glass ceiling. Information, diagnosis of personality disorder not shared [55]
Survivor roots	Richards et al. [3], Adame [1] Foundational nature of survivor identity. Jones [53], Cameron et al. [20] Screaming in a milk bottle [55]

### The positions of identity

Five identity positions became apparent; each of these is described below in Table 4.

### Service user and survivor identities

Service user and survivor identities were common across all studies. The data identified service users, survivors, drug users and experts by experience. These identities were separate to the service provider and held less power, control, and respect. Cameron et al. [20] reported that services perceived the service user or disabled person as the one with a problem. Their identities are also perceived as “limiting” (Newcomb et al., [23], p 2). DeRuysscher et al. [21] also found that service users were defined and overshadowed by their service user identities. Service user involvement work provided the opportunity to transform these identities and move beyond the stigma associated with them to more positive identities not rooted in deficit [54]. The idea of role reversal, where the service user became the provider through the expert by experience role, changed the power differentials and enabled service users to be seen as people to learn from [52].

### Professional identity

Across the 13 studies the professional identity of lived experience researchers and providers was constructed. This consisted of having skills and competences to effectively carry out these roles [20] and motivation to combine existing competences from personal lives into professional roles [52]. Richards et al. [3] found those with professional identities were seen as knowledgeable, and competent, with more power than those with just service user identities. Peer workers were focused on developing professional identities which were legitimized through training [14] and appropriate titles [52]. There were allowed and disallowed characteristics, such as it “not being acceptable to become angry” [3] p 6], “having everything together” and “never having a bad day” (Wilson et al. [22], p363). Jones [53] reported, being an expert by experience required that one must communicate articulately and clearly. Cooke et al. [55] find that the development of a professional identity shifted service users to feel as though they have greater value and power. Within these studies, it was reported that service user researchers and providers were more likely to want to convey their expertise as people with knowledge who were skilled at their jobs to detract from their stigmatised service user identities.

### Integrated identities

Integrated identities were discussed within the research as individuals holding service user and professional identities simultaneously, and this was problematic and conflicting for the individual. There were differently held beliefs of whether integration was useful or not. Richards et al. [3] found within an “integrated” identity, participants drew on all their identities to inform their practice, but this was rarer than the unintegrated identity as it was more difficult to accomplish. Newcomb et al. [23] found when academics shared their lived experience in professional contexts it reduced stigma and provided student healthcare providers with examples of how to integrate their own lived experiences. However, research [1] found integrating lived experience excluded them from being accepted by colleagues within the profession and by other survivors of the mental health system. The idea of integration was spoken of as embodiment but was emotionally burdensome in peer researchers [25].

### Unintegrated

This theme addressed the issue of being unable to hold identities of service user and professional simultaneously. Richards et al. [3] reported the “mad man versus someone who got a reputation for being highly professional they’re worlds apart unfortunately.” [3, p7]. Service user and professional identities were understood as separate and either good or bad. Research [1] found that, despite mental health professionals having personal experience of mental illness they were likely to keep that hidden. Cooke et al. [55] also find that being perceived as the one with lived experience in the room was conflicting, leading to a reluctance in wanting to share. Newcomb et al. [23] reported this was due to stigma and fear over queries over fitness to practice. This fear stopped some peer workers from seeking help when they relapsed [22]. Cameron et al. [20] also identified the conflicting positions experts by experience occupy, where in one context they are sources of knowledge, and other contexts as consumers of care. The service user representative role required service users to share their stories but with an expectation to separate the emotion from storytelling, to create distance from the service user identity to support learning from experience that could be tolerated by healthcare professionals [25, 52, 53].

### Liminality/ambivalence

The concept of liminality [37] is applied by Simpson et al. [14] and Faulkner and Thompson [25] to describe the experiences of peer workers who occupy a space in between being a service user and professional. The role ambiguity through occupying in between identities meant that it was difficult for peer workers to understand how they should interact with the people they support and the teams they work in. There was a lack of understanding whether they were friends or peers or a different dynamic. This had consequences for how others perceived them, and unclear expectations of the role and services they provided. This identity ambiguity led to differences in respect and power associated with these roles. This posed similar dilemmas to the lived experience researcher holding ambivalent identities [25].

### The EMERGES framework

**Fig. 2:**
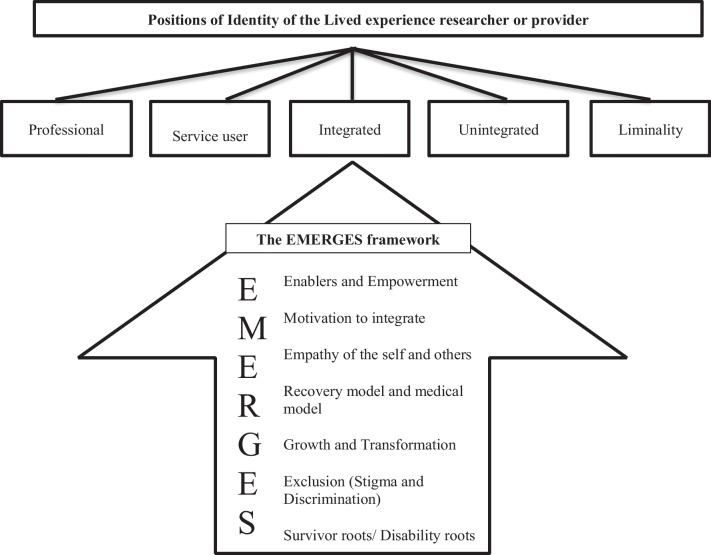
A visual representation and summary of findings in the review

The data in the studies informed the EMERGES framework where 7 core themes related to identity development were found, encompassing: Empowerment, Motivation, Empathy of the self and others, Recovery model and medical model, Growth and transformation, Exclusion and Survivor roots as demonstrated by Table 5. The framework is illustrated in Fig. 2. This is presented in reverse and ascending order starting from survivor roots through to enablers and empowerment replicating the journey that the current research suggests lived experience researchers and providers go through to develop their emerging identities.

### Survivor roots

Adame [1] found the survivor identity were the roots and drivers of their need to work in the system. “The survivor part of me is what gets me out of bed each morning, and thinks that what I’m doing is important, and meaningful, and really needed…Like this background motor, I guess. It’s its own string of conviction, this motor, this energy that’s all in the background.” [1, p327] Jones et al. [53] found that participants were likely to draw on their acute struggles of lived experience in their roles. Toikko [52] identified how having lived experience of mental distress was the foundation to becoming an expert by experience. A parallel identity to that of survivor roots was that of disability roots, and this was the source of motivation to challenge and disrupt the system which is disabling [20].

### Exclusion/stigma and discrimination by services

This theme covered how lived experience researchers and providers felt they must hide their lived experience due to queries over competence and fitness to practice. [1, 23]. Service providers also chose who they listened to, and involvement could be tokenistic, recycling oppression lived experience researchers and providers experienced in contexts where they were service users [20]. Certain voices were excluded that were more chaotic and less professionalized [53]. Cooke et al. [55] also identify how the label of personality disorder is shaming and stigmatising, and diagnosistic practices operated in an inclandestine way excluding their involvement. Exclusion and alienation of those from minority ethnic backgrounds within these spaces was also discussed [25]. Hill et al. [56] also related service user and carer involvement to a need to “break the glass ceiling” as staff were seen to hold the power and control the agenda.

### Growth and transformation

This theme encompassed experts by experience and co-researchers discussing effects of involvement leading to a metaphorical growth and transformation. “Seeing everybody still ‘fighting for it’…the enthusiasm is more than ever… these are different people to the ones three years ago, I’ve been able to watch my teammates blossom!” [54, p646]. Richards et al. [3] found the impact of these roles resulted in positively framed identities, facilitating recovery. It enabled individuals to have alternative, additional identities where the service user identity did not overshadow them. “So, it’s not the most central thing anymore, that you’re a mental health patient, but rather that you are a lot more as well.” (Toikko, [52], p303). Hill et al. [56, p 9] also found the theme of “The person you see now is not the person I was.” This growth and transformation was related to the emergence of a professional identity, moving further away from the service user identity Cooke et al. [55].

### Recovery model versus medical model

This theme found the recovery model was a facilitator in changing mental health identities to be seen as recovered. However, both the recovery model and medical model could both empower and disempower. Across the studies identities of lived experience researchers and providers were rooted in and influenced by these models. Despite models such as the social model of disability not locating the problem within the service user, the lens in which service providers worked “gets shifted back to medical model approach” Cameron et al. [20, p 1323] influencing identities to be seen as disordered. Richards et al. [3, p 10] found that those who drew on a “personal recovery” had more positively framed identities because it placed less emphasis on being “stuck”. Adame [1] identified an alternative discourse regarding the medical model, some service users found it helpful, and when the provider challenged the service users’ alignment with the medical model, or of diagnosis, it was invalidating to how service users understood themselves and their experiences. Cooke et al. [55] felt the process of diagnosis was disempowering but became empowering once service users understood that the damage that came from diagnosis came from service providers. Hill et al. [56] also found that relations between service users and carers within involvement groups required a management of power dynamics.

### Empathy of the self and others

This theme covered how the experience of being a lived experience researcher and provider led to an understanding of the self and others. Service user representatives found sharing stories of personal experiences turned them into common shared experiences of distress, enhancing empathy and reducing stigma. The PSW discussed the importance of being ‘one of them’ and able to ‘get it.’ Simpson et al. [14, p 665]. Richards et al. [3] and Newcomb et al. [23] also found that personal experiences of distress enabled better understanding of those they worked with. Providing a social domain in which individuals shared their experiences as co-researchers meant they felt, understood, and better understood others [52–54, 56]. In relation to each other they also felt like they had similar experiences and a sense of “group survivorship.” Hill et al. [56], p 6]. Richards et al. [3, p 9] also identify a similar group identity through the idea of a shared “personhood” and Cooke et al. [55], p 239], found it useful to work with others who are similar like a “band of brothers.” Adame [1] found empathy of the self and others was impacted by overidentification, blocking a therapist’s understanding of those they support. This meant that these roles sometimes supported or hindered understanding the self and others.

### Motivation to integrate

Adame [1] and Newcomb et al. [23] identify that lived experience providers were motivated to make a difference to others, due to their own lived experiences and wanted to prevent others experiencing the same injustices of the system. Positive experiences of services were motivators for becoming lived experience researchers or providers and modelling this experience in their own practice. Additionally, motivations to apply professional knowledge to better understand their own experiences was important [3]. Cameron et al. [20] found a motivator to continue in the work as a service user representative was to purposely disrupt the power dynamics in these contexts. There was a need to move beyond the service user identity [54] and change their own narratives to more positively framed senses of self with a purpose in life. Faulkner and Thompson [25] also identified that it was not simply enough to be working as lived experience researchers but to actively be integrating their lived experience into their work. Cooke et al. [55], p 239] also find that expert by experience roles were motivated in making use of past struggles “It almost comes worthwhile because you can almost see you’re doing something with it.”

### Enablers and empowerment

The lived experience researcher and provider role enabled moving beyond the service user identity [49]. This was influenced by learning and combining new and existing skills [14, 20, 22, 52, 53, 56], and contexts situated in the recovery model, gave hope [3]. Toikko [52, p 303] found that being an expert by experience led to an orientation towards the future. They were empowered after involvement [53] and through being listened to, heard, and meaningfully involved [20]. It also gave meaning and purpose through “planting a seed” (Cooke et al. [55], p 240). Hill et al. [56] also found that meeting challenges, resulted in self-belief by having control over decision-making. Activism, social change, politicised identities, and positive identities were developed because of these roles and were thought to facilitate recovery [1, 20, 53, 55, 56]. However, some providers maintained there were fewer opportunities for those with drug user identities that were disempowered in their roles due to stigma and the permanence of a service user identity [22].

### Stage 3: checking the robustness of the synthesis: reflections by alison bryant, service user advisor

“I am in awe of the EMERGES framework and thoroughly relate to the themes and how my experience is integrated into those themes.”

The robustness of the synthesis was checked by each member of the research team (VG, PF, LG, BG & CE) and researchers at McPin Foundation (RT & TM). AB, service user advisor uses the EMERGES framework to reflect on her lived experiences and evidences its utility as a reflective tool.

**Survivor roots** “*My long history of mental health presentations has defined me at every stage of my life. My experiences of services have been very varied, adding to the burden of my lived experience. Clinical psychology, and specifically mindfulness, has helped me to survive and be able to acknowledge that I have survived, and is now deeply meshed as part of my lived identity.*

**Exclusion/Stigma and Discrimination**: *I know holding my lived mental health experience and identity as being valuable to others (let alone myself) has been a hard road to travel. At times, my own perception of stigma initiated feelings of exclusion, but also, I acknowledge that I have ‘lost’, through smoke and mirrors, some of my history and identity when I realised this was neither valued nor accepted if not absolutely rejected. When the presence of imposter syndrome arises regarding my identity whilst working alongside academic or professional identities with no lived experience, I try to comfort myself that this is to be expected and to work towards reducing my feelings of exclusion.*

**Growth and Transformation**: *I and my family know how much my identity has been shaped by my involvement as a service user/provider. Both self-stigma and societal stigma have been a lens through which I have viewed my lived experience of mental health, this view having now been reframed in the context of my service user involvement. These experiences validate me and acknowledge my voice is heard. My knowledge sharing has empowered me so much, to the extent that sometimes I consciously listen to my voice that was once so subdued with a sense of surprise and ownership previously lacking.*

**Recovery model versus medical model**: *My clinical psychologist, in presenting me with the then novel concept that my experiences would be valued by others, was instrumental in me taking on the role of service user/provider. My initial involvement was at times bewildering, often surprising, but allowed my identity to develop bit by bit over time. My recovery from psychosis has been reinforced through my service user identity and involvement, but I am all too aware that there are periods when my mental health is less stable, and my service user involvement may be seen as less productive or useful. This presents me with an insurmountable hurdle to achieving full involvement unless, in the future, the “goal posts” are shifted with mental health adjustments to better support service user providers.*

**Empathy of the Self and others**: *My service user involvement was a seed planted by my clinical psychologist that related to part of my identity which had always been at the forefront, and a heartfelt wish that others never had to go through the experiences in life and in managing mental health that I had done. Being able to demonstrate as a service user provider to those in training the reality of my lived experience helps shape them as practitioners. Sharing with other service users, identifying with them, and offering support and solidarity through the challenges of shared lived identity is very empowering.*

**Motivation**: *For many years, my sense of self and identity had been eroded by the effort of constantly battling my mental health and despair at the impact on my quality of life and that of my family. My ability to be confident, to interact socially, my sense of self-esteem and sense of purpose had become lost in the struggle to become well. My most recent contact with services was a key factor in my recovery, and the incentive generated because of the therapeutic alliance with my clinical psychologist to help others was an overwhelming driver in my journey to recovery and new identity. My fractured self was pieced together in my pursuit of my newly formed service user identity.*

**Enablers and Empowerment**: *I had over time lost sight of skills or abilities I had held as part of other identities. Becoming a service user/provider allowed me to revisit those identities, to tease out what would sit alongside my mental health lived experience, to empower other service users, health professionals, trainees and ultimately myself. I now have a new perspective on my experiences gleaned from this new vantage point. I once had only a tiny seed of hope, now this has blossomed giving me a new sense of identity, purpose and direction*.**”**

## Discussion

The review aimed to understand how the process of working as lived experience researchers and providers in mental health, education and research settings impacted identity and to develop a conceptual framework. The framework identifies five different positions of identity: Service user, Professional, Integrated, Unintegrated and Liminal identities and details influencing themes of the EMERGES framework consisting of Enablers and Empowerment, Motivation, Empathy of the self and others, Recovery Model and Medical Model, Growth and transformation, Exclusion (Stigma and Discrimination) and Survivor roots. The EMERGES framework is a novel conception and has common themes of emotion and power running throughout, with some overlap between themes.

### The positions of identity

The service user position is characterised as being disordered, limiting and considers the individual as the one with the problem. This is consistent with research in mental health settings, where illness identities are detrimental to hope and recovery, resulting in poorer mental health [57]. The lived experience researcher or provider is expected to control their emotions and keep a distance from their illness, detracting from diversity and representativeness of service users who are chaotic or suffer from severe mental illnesses, influencing the type of identities within these roles [58]. The service user has to switch between positions, for example, having to move between service user and expert by experience, where there are different levels of power, and control in decision-making, requiring negotiation [2].

Professional identity was reinforced through training and labels used to describe them, giving them the skills and competences to work in their roles. This is consistent with, the Academy of Medical Royal Colleges [59] that identify skills and knowledge to perform a professional role are key to developing a professional identity. Mayer and Mckenzie [60] also find professional identities of experts by experience are influenced through interactions with experts by qualification and through performing these roles.

The Integrated identity was characterised by sharing lived experience both in research and clinical practice. Beames et al. [61] find the integration of lived experience in all stages of research supports meaningful research and outcomes. Arroll and Allen [62] find self-disclosure results in greater therapeutic rapport and empathy. However, Bray [63] identifies the risks of self-disclosure and how it de-centres the service user. Alternatively, the peer support worker role requires them to work with patients through a shared experience of distress, but they do not necessarily need to disclose as there is already visibility of lived experience. Sharing of lived experiences by professionals is likely to de-stigmatise the idea of mental illness [64, 65]. Integrating lived experience in professional roles is related to being an authentic version of the self. Research into authenticity suggests that when we are authentic it is better for our health and wellbeing [66], providing support for the benefits of lived experience roles.

The Unintegrated identity of the service user and professional identified how these identities were conflicting and could not be held simultaneously. Research suggests that experiential knowledge comes predominantly from the expert by experience, suggesting learning about experiential knowledge cannot come from mental health professionals. Additionally, professional knowledge is better assimilated when it comes from healthcare professionals as opposed to those with lived experiences [16]. This is explained by epistemic and hermeneutical injustice which poses limits on where knowledge is learned from [15]. Lived experience researchers and providers are also required to separate emotion and maintain professionalism in their roles. They must convey “affective intensity, while not spilling over into uncontrolled illness” (Naslund et al. [67]: p10). Researchers and professionals with lived experience are also impacted by stigma in the profession of lived experience that may determine whether they integrate lived experience in their research or clinical work.

Further to this, an unintegrated or integrated identity largely depends on the role and the level of visibility of lived experience within that role. For example, mental health professionals have a sense of privilege as they can choose when or when not to disclose their lived experiences [68], as this is not the purpose of their role. In contrast to this, experts by experience or peer workers are specifically employed to voice and embody lived experiences. It is also acknowledged by some experts by experience that there is a choice over owning different identities at different times by choosing when to wear the lived experience hat [13]. Although for those employed in lived experience roles this might not always be possible as there is an expectation from others that they must work from an experiential lens. Alternatively, those in peer roles embody lived experience, but there is control over articulating the specifics of this experience as they operate in a relational way, connecting through common experiences of distress that do not necessarily depend on disclosure. Although, due to the visibility of their lived experience, research shows peer workers are more likely to disclose, encourage and elicit disclosures from patients and other healthcare professionals, consequently increasing the visibility of lived experience in a clinical context [69]. These examples across these different groups serve to articulate the distinction between these different roles and the extent to which they can integrate lived experiences.

The process of liminality [37] described the identity of peer workers and researchers [14, 25]. Wu et al. [70] suggest that Liminal spaces negatively impact the mental health of individuals occupying this space. Warner and Gabe [71] identify how mental health social workers find it difficult to work with mental health patients who occupy liminal spaces as they are difficult to understand and support. This can also translate to the way lived experience researchers and providers are understood and worked with in clinical practice and research, by colleagues. Although, anecdotal evidence suggests those in lived experience roles are likely to have a better understanding of their own identities. Whereas those they work with will often perceive them as occupying liminal identities. These findings can support service providers and colleagues to better understand those with liminal identities and enable better team working. It also identifies how these roles have an emotional burden on those performing them.

### The EMERGES framework

Outlined below are the seven core elements of the EMERGES Framework found as a result of the systematic narrative review. Each element is considered and discussed in relation to the evidence base and how lived experience researchers or providers can be better understood and worked with in mental health, research, and educational settings.

### Survivor roots

This theme found becoming a lived experience researcher or provider was rooted in the history of being a survivor or service user of the mental/health system. The experience of trauma or iatrogenic harm from services can influence changes to identity. Through the process of having positive or negative experiences of services may be formative to self-identifying as a service user or survivor differently. Wallcraft et al. [72] identify the diversity of perspectives within and between service users and survivors but identify shared motivations to improve the mental health system. The intersectional [41] influence of lived experience and professional aspects to the role means that this researcher or provider has more complex needs and requires greater support.

### Exclusion/stigma and discrimination by services

Stigma and discrimination were shown to negatively impact disclosure of lived experience and health-seeking behaviour and this is seen in wider contexts [73, 74]. The review team’s own observations find service user involvement is typically made up of white service users and is unrepresentative of the population which may be symptomatic of exclusion in the mental health system. There are also them and us divisions between lived experience researchers and providers and those they work with. For example, knowledge of stigmatised diagnostic labels, such as personality disorder, affects how experts by qualification perceive and work with them [75]. There are also divisions between different lived experience researchers and providers [1], for example, experts by experience and mental health professionals with lived experience, meaning different lived experience researchers and providers do not belong to the same identity.

### Growth and transformation

The review found a consistent theme of growth and transformation. This links to a broader body of evidence within the literature on how service users or survivors of mental or physical disability experience post-traumatic growth [76]. Theoretically driven research metaphorically likens the effects of service user involvement to growth and transformation [38] and research finds the expert by experience role results in transformative effects [77, 78]. Some lived experience researchers and providers also have a romanticised perspective and find transformative effects in identity following the experience of psychosis [33]. Schneider et al. [79] find non-white people and those with serious forms of distress are more likely to experience greater post-traumatic growth, suggesting the trajectory of growth and transformation of lived experience researchers and providers may be variable.

### Recovery model and medical model

The wider literature identified the recovery model was more likely to lead to feeling more hopeful and move individuals further from the service user identity [80]. The recovery model arguably has a negative side that promotes a certain journey for service users, modelling ideas about competence, expertise and health outcomes that reduce the ideal service user to someone that is recovered [81]. This limits the type of individual in lived experience researcher and provider roles, reducing the representativeness and authenticity of service users. The recovery model ironically detracts from the service user identity. In contrast, the medical model reinforces the service user identity, positioning the service user as in need of help, as ill or disordered [82]. The context and models in which individuals are situated in can influence the way individuals conceptualise their own experiences. This aligns with social constructionism epistemology [83]. The recovery model, medical model and social disability model are pervasive in the sector and explain findings in the review and how lived experience researchers and providers’ differently construct their identities based on the models they identify with.

### Empathy of the self and others

This theme found how sharing experiences within a social domain were used to connect with and understand others. The social identity of the lived experience researcher and provider role provided a sense of belonging to an in-group where we share similar values, beliefs, and experiences, supporting the formation of a social identity [34]. The historical exclusion of this group of people in society means the role enables them to have a sense of belonging. Hawkins [84] suggests a desire to tell others about our own experiences becomes a desire to help others and this is a motivating factor in integrating lived experience. The process of lived experience researcher and provider identities may mean they better understand the people they research or work with and make them better person-centred practitioners [85]. However, issues relating to transference and countertransference can negatively impact understanding others through the projection of one’s own lived experiences.

### Motivation to integrate

This review identifies the idea of motivation to integrate lived experience and professional identities and hold them simultaneously. This motivation aligns with wider mental health contexts, for example, the division of Clinical Psychology [7] released guidance on how trainee psychologists can integrate their lived experience into their work and training. This suggests the lived experience researcher or provider and mental health training are in alignment regarding motivations to integrate lived experience. This is likely influenced by changes in policy (Department of Health, DOH, [86–88] and the service user movement [5].

### Empowerment and enablers

The review found the idea of empowerment through lived experience researcher and provider work, and this may occur through a social justice motivation [89]. Through lived experience researcher and provider roles that are politically motivated, formed of activists and advocates means they are moving away from individual motivations to make a difference to a collective motivation to make a difference for others like themselves. This may be governed by social identities [34]. Belonging to a social identity is likely to result in the health and wellbeing of members in the group, strengthening the group and empowering it and advocating for it. This can be explained through the social cure phenomenon in social identities [90]. Further to this, reaction against out-groups provides a motivation to disrupt and challenge other social identities such as Psychiatry or Psychology.

### The EMERGES framework and links to other frameworks

The EMERGES framework conceptualises the identities of lived experience researchers and providers and builds on previous frameworks in other contexts, offering a novel way to understand identity. There are parallels with findings from the CHIME framework, which is made up of themes of Connectedness, Hope, Identity, Meaning, Empowerment [46]. Although the CHIME framework is critiqued as being overly optimistic and positive, and other researchers argue it does not account for difficulties that service users experience, advocating for an addition of D to the CHIME framework representing difficulties [91]. In contrast, the EMERGES framework explicitly highlights the exclusion lived experience researchers and providers experience which is undermined by the retrospective addition of the D in CHIME-D framework. There is also overlap with Emery’s literature review [47] of service user involvement, finding themes of empowerment, recovery, and identity, giving validity to this review. Ng et al. [92] find in their conceptual review of psychosis and growth and transformation the acronym of PROSPER, Personal identity and strength, Receiving support, Opportunities and possibilities, Strategies for coping, Perspective shift, Emotional experience, and Relationships. The EMERGES framework encompasses similar themes to these frameworks but specific to lived experience researchers and provider identities, offering a novel and accessible way to understand them.

### Strengths of the review and framework

The review identifies the novel EMERGES framework which can be used as a reflective tool and has practical applications both within research and clinical settings with the heterogeneous population of lived experience researchers and providers. The framework was co-created with a multi-disciplinary team, including lived experience researchers and providers, at every level within the review, adding to the robustness and validity of the findings and additionally, peer-reviewed by researchers at McPin Foundation. Quality appraisal was also carried out independently by three different PhD Psychology students, with different expertise in lived experience and research methods. The methodologies within the studies were informed by a wide range of philosophical approaches that contributed to this synthesis. The research questions and aims of the studies differed slightly and so their synthesis may not represent each individual study’s findings cohesively, but instead, the review identifies common themes across all. These are informed through a range of perspectives and philosophical underpinnings that supports the robustness of the synthesis. The framework has also been reviewed by researchers in other settings who suggest its value and application in mental health settings more generally, evidencing its versatility and wide-reaching impact.

### Limitations of the review

It is acknowledged the review groups together different populations such as experts by experience, lived experience researchers, peer workers and mental health professionals with lived experience and treats them as a homogenous group. There are differences across these groups and subtle nuances that the synthesis may not have identified. However, through identifying the relationships between these groups, the review develops a broader synthesis and framework that is informed by each role and their commonalities that has explanatory power to help us better understand the identities of lived experience researchers and providers more generally. This may counter the ‘Them and Us’ divisions that often exist between these groups and others, by identifying their similarities. The review also did not explore the grey literature, or literature on ethnicity, LGBT, and gender identity and this may have cast further insight into identity formation, but this was not a focus of the review. Most studies within this review failed to discuss the lack of diversity within lived experience researcher and provider roles. Only one paper [25] addresses this issue explicitly, but this study’s sample size was mostly white, meaning it is difficult to draw meaning from such conclusions. The reviewed studies highlight how the field is limited to lived experience researchers and providers who can communicate their experiences and manage their emotions with competence and professionalism. This excludes those with severe mental or physical disabilities and those with learning disabilities. The carer voice and their work as experts by experience or providers is also largely missing from the studies included within this review, only, Hill et al. [56] included carers within their study. A proportion of the studies within the review are complex and academic in nature and may be difficult for lay people to understand, suggesting that the people who can benefit from the research may not due to lack of accessibility. This meant that the review team were mindful of those who would benefit from reading the review and were motivated in communicating this in an accessible way. It is also acknowledged the field of lived experience work is referred to in a diverse set of ways nationally and internationally and so the search strategy may not have captured all research in the area. There is also a lack of literature exploring the effects of lived experience researchers and provider roles on identity, evidenced through only thirteen papers in this review, and so there is a recommendation for adding to the evidence base.

## Conclusion

This review elucidates the identities of lived experience researchers and providers in mental health, education, and research and gives greater clarity to these identities that are sometimes not understood by themselves or the people they work with. The EMERGES framework can be used as a reflective tool to better understand those is these roles and support effective team working. The review highlights how lived experience researcher and provider roles are performed by individuals with certain characteristics such as those who are professional, articulate and those who can separate and integrate, when appropriate, their lived experiences. However, people who do not have these characteristics, or people from ethnic minority backgrounds, in addition to those with severe and enduring chronic mental, physical and learning disabilities, are often excluded from these roles. Furthermore, it is evident that carer involvement in these roles is also underrepresented. This clearly limits the identities of those in these roles. Through the integration of more underserved communities in lived experience roles can lead to a depth of experience that can be drawn upon, leading to growth and transformation in the sector. However, the integration of lived experience within these contexts is limited, due to stigma and discrimination. This limits self-disclosure and health-seeking behaviours which may be due to the professionalisation of these roles. Therefore, those working with lived experience researchers and providers need to be aware of their support needs which can be guided by the practical application of the EMERGES framework. There is also a need to integrate lived experience to be authentic and also a motivation to promote social justice in the mental health system. Through the process of sharing lived experiences supports others to listen and learn from them and supports greater empathy of one’s own distress and that of others.

Through the process of performing these roles and through moving through the stages of the EMERGES framework leads to identity development. In some cases, the stigma of mental illness, or disability permanently marks the identity of lived experience researchers and providers, affecting their prospects and the lens through which they are viewed. However, the general trend among the literature highlights how lived experience researcher and provider roles moves them beyond the service user identity. This consequently transforms those with stigmatised identities to enabling and empowered identities, facilitating recovery.

## Supplementary Information

**Additional file 1. Stage 1:** Preliminary synthesis and data extraction table.

## Data Availability

All data generated or analysed during this study are included in this published article [and its supplementary information files]. The preliminary synthesis, detailed quality appraisal of each researcher and table of original sources into a framework can be found on open access dataset found here: https://osf.io/xnt2p/. A pre-print of the systematic review can be found here: Gupta V, Golding L, Eames C, Greenhill B, Qi R, Allan S, Bryant A, Fisher P 2022 https://psyarxiv.com/gjh2c/

## Abbreviations

HCPC: Healthcare professions council
BPS: British psychological society
GTICP: Group of trainers in clinical psychology
RCPsych: Royal college of psychiatrists
NIHR: National institute of health research
NSUN: National survivor user network
SRN: Survivor researcher network
SOL: Shaping our lives
SPIDER tool: Sample, phenomenon of interest, design, evaluative, research type
JBI: Joanna Briggs institute
PSW: Peer support worker
DOH: Department of health
EMERGES framework: Empowerment and enablers, motivation, empathy of the self and others, recovery model and medical model, growth and transformation, exclusion, survivor roots
CHIME framework: Connectedness, hope, identity, meaning, empowerment
PROSPER: Personal identity and strength, receiving support, opportunities and possibilities, strategies for coping, perspective shift, emotional experience and relationships
LGBT: Lesbian, gay, bisexual, transgender
