# Supplementary material for: Understanding the identity of lived experience researchers and providers: a conceptual framework and systematic narrative review
Source: Res Involv Engagem. 2023 Apr 24;9:26. doi: 10.1186/s40900-023-00439-0 (PMC10127294; doi:10.1186/s40900-023-00439-0)
Supplement: Supplementary file 1 — Additional file 1. Stage 1: Preliminary synthesis and data extraction table. [file 40900_2023_439_MOESM1_ESM.docx]

| **First Author (Year)**  **Additional file 1: Table S1 Stage 1 A preliminary synthesis (Insert p12)** | **Aims** | **Study population** | | | | | **Method** | **Summary of findings and core themes relating to identity** |
| --- | --- | --- | --- | --- | --- | --- | --- | --- |
| Adame (2011)  USA | To explore the implications of being a survivor and mental health professional and the impact on identity | 5 participant interviews of therapists who are also survivors. | Age not reported. | Gender not reported. | Ethnicity not reported. | Type of mental illness not reported. | Holistic content analysis. | Themes identified the foundational nature of the survivor identity. Risks and benefits of self disclosure of lived experience were identified. They find they have a greater understanding of their patients but also identify a risk of overidentification. Us and Them divisions were found between survivors and professionals. Differences between psychiatric and psychological models was also discussed. |
| Newcomb et al (2017)  Australia | To explore how social work and human services students with service user experience integrate their lived experience in training. | 20 Undergraduate Social work and Human services students who had accessed services. | Age not reported. | Gender not reported. | Ethnicity not reported | Type of mental illness not reported. | Inductive thematic analysis | Five key themes; motivation to enter social work and human services due to personal experience as a service user and a motivation to help others like themselves. There was also the idea of positive role modelling and supporting others in ways they have been supported. The idea of being unfit to practice stopped disclosures. Both service user and service provider identities were not easily integrated. |
| Richards et al (2016)  UK | To explore identity of mental health professionals with mental health service user experience | 10 participants who were mental health professionals with experience of being a service user. | Age not reported. | 5 male and 5 female. | 7 White British, 2 Asian, 1 South African. | Type of mental illness: depression, suicidal ideation, paranoia, schizophrenia, Bipolar disorder, Psychosis, Anxiety and Bulimia. | Discourse analysis  A social constructionist epistemology | Themes found included separate unintegrated and integrated identities of “Professional” and “Patient.” Discourse on Professional identity attributed power, knowledge, and expertise whereas Patient identities were perceived as stigmatised, influencing a lack of disclosure. Integrated identities led to a new sense of self. Integration of mental health patient identities and professional identities gave new skills and power. |
| Simpson et al (2018)  UK | To explore the occupational and liminal identities of peer support workers | 8 Peer support workers and 13 Service users | Age range between 20 and 55 years old | 5 male and 3 female peer support workers  8 male and 5 female service users | White Irish, White other, Bangladeshi, Black Caribbean, White British, White other, Black British, Black African, Mixed, Black/British African and Black European | Service user mental illnesses reported but not peer support workers  Depression, Paranoid Schizophrenia, Psychosis, Unknown, Schizoaffective disorder | Thematic analysis with deductive application of Liminality theory | The analysis revealed lived experience influenced service users to better connect with peer support workers who were like them and understood them and who were able to model recovery. The role supported in fostering a new identity and motivation to move past the service user identity. Occupational training and learning new skills legitimised occupational identity. Relationships with peers had positive effects and likened to a friendship but were also confusing. Peer worker identities were somewhere in between service user and professional, leading to liminal identities. |
| Wilson et al (2018)  Canada | To identify challenges of peer workers and provide recommendations based on emerging themes of identity | 11 peer workers and 6 informants were interviewed.  Peer workers who are previous drug users supporting drug users in harm reduction. | At least 18 years of age | 4 Male informants and 2 female informants  4 male and 7 female Peer workers | Ethnicity not reported. | Previous or current drug users, but other mental or physical disabilities not reported | Grounded theory analysis and symbolic interactionist analysis. | The drug user and peer worker identities were perceived as distinct. The identities of peer workers relied on the idea that they were in recovery and no longer using drugs, impacting disclosure and health seeking behaviour. The second theme was that of Triggering, where the role often made it difficult for the peer worker to remain well. There was pressure to perform, and they held themselves to a certain expectation they perceived a peer worker should be held to. The role also meant that they were stuck with the stigmatising drug user label in their peer working roles. |
| Toikko (2016)  Finland | To understand the learning process behind becoming an Expert by Experience. | 12 participants Mental health service users that become experts by experience | Age range 28 - 57 years old. | Gender of participants unclear. | Ethnicity not reported. | Type of mental illness not reported. | Thematic analysis | Four different themes. 1. Creating distance from experience: This meant having distance from the emotion of mental health experiences through storytelling. 2. Sharing experiences with peers and friends: this process describes how listening to the stories of others and sharing experiences facilitated recovery 3. Combining experiences with existing competences and skills learned through previous professional roles or personal experiences were formative to identity. 4. The roles also led to developing an orientation to the future. |
| Jones et al (2020)  Finland | To explore the identities of service users who become Experts by experience. | 13 participants, Service users and/or Carers that are Experts by experience. 11 of which has mental illness. | Age range 23 - 62 years old | Gender not reported. | Ethnicity not reported. | Type of mental illness: Bipolar disorder, Schizophrenia, Psychosis and Depression. | Thematic analysis | Being an expert by experience helped to reframe lived experience through constructing and sharing stories, creating distance from it. Illness experiences were sources of motivation to change their narrative to recovery with opportunities for self-discovery and personal development. The illness identity was constructed as being in the past and recovered identities in the present. Professional identities were constructed through learning new skills and combining them with existing skills to elevate their status. Social connections and collective shared grievances motivated politicised identities. |
| Hutchinson et al (2013)  UK | To describe the identities of service user researchers and the effects on identity. | 6 Co-researchers interviewing 30 services users. Mental health service users and co-researchers that are also mental health service users. | Age range 30 - over 65 years old | 5 Female and 1 Male | Ethnicity not reported. | Type of mental illness: Bipolar Disorder, Depression, Anxiety Disorder, Postnatal Depression and Eating disorder. | Interpretive Phenomenological analysis | Two themes were found within the research of co-researchers connecting with the service users’ stories. There was an idea of unrestricting lives and reciprocity. This theme represents connections with others and feeling belief in themselves through belief from others in you. Hearing others’ stories enabled empathetic connections. The second theme was reframing the illness narrative, where the process of hearing other people’s stories of mental distress humanised the people behind the diagnosis. The medical model made individuals feel inferior but involvement as co-researchers empowered them. |
| Cameron et al (2019)  UK | To understand the impact of service user involvement on identity. | 22 disabled individuals who are service user researchers | Age range  Under 40 to 70 years old | 12 women, 9 men and 1 nonbinary. | Ethnicity not reported | Type of impairment: sight loss, being deaf, acquired brain injury, cognitive impairment, learning disability, mental illness, non-epileptic seizures, multiple sclerosis, cerebral palsy, mobility impairment, wheelchair users and Carers | Thematic analysis | Involvement as experts by experience was found to give them more power but was sometimes tokenistic. The same individuals when perceived as service users held different levels of power, respect, and value. Good outcomes and meaningful involvement lead to empowerment, purpose, feeling valued, and validated, gaining new skills, knowledge, and social connection. However, service providers unintentionally oppress experts by experience through reverting back to medical model approach that diminishes identity and power of experts by experience. |
| De Ruysscher et al (2019)  Belgium | To understand the recovery process of a patient who becomes a peer worker. | 1 Peer worker and expert by experience | Age not reported. | Gender 1 male peer worker and 1 female academic researcher. | Ethnicity not reported. | Type of mental illness: Paranoid delusional psychosis, drug addiction | Bricolage method with a thematic analysis | Four themes found including 1) Life rebuilding encompassing ideas of living a meaningful life with responsibilities such as having a job, hobbies. Social circles were seen as essential in rebuilding life because of stigma and discrimination, 2) The patient identity was seen as salient when undergoing treatment and stigmatised. 3) Continuity of care, and person-centred care were essential to effective service provision and recovery. 4) The role of drugs theme explained that it was difficult to disclose and seek help when relapsing due to the expectations of the peer worker role and stigma of drug use. |
| Faulkner & Thompson (2020)  UK | To identify the emotional impact of lived experience work, effects on identity and challenges and benefits and methods to support these researchers. | 10 lived experience researchers | Age not reported | Four male and 6 female. | Ethnicity, 8 White, 2 South Asian. | People with lived experience at various levels and stages in their roles. One had a physical disability. | Thematic analysis | Themes found the idea of negotiating identities of lived experience researcher in their roles and showed there was emotional burden and labour associated with embodying lived experience roles and they found experiences of alienation and exclusion in research and how they had to navigate the bureaucracy of the systems and they identify support strategies within their key findings. |
| Hill, Tickle & DeBoos (2021)  UK | To explore personal effects of involvement as service users and carer representatives within clinical psychology training | 14 service user and carer representatives | Ages 25-79 years old | 6 Male  8 Female | Ethnicity White British, British, Asian British, British Indian, Mixed, Italian, German Syrian | Type of mental illness or disability not reported but type of services accessed reported | Thematic analysis with critical realist approach and deductive application of psychological theory | 5 themes including Theme 1: Environment determines sense of safety including the influence of supportive relationships in the team and by staff. Theme 2 was about “meeting challenges” and feeling empowerment and a sense of belief in the self. Theme 3 was about gaining a sense of purpose through being listened to and making a difference. Theme 4 was about “The person you see now is not the person I was,” and Theme 5 was about “breaking the glass ceiling” and a sense that involvement was controlled by staff. |
| Cooke, Daiches & Hickey (2015)  UK | To explore the narratives of experts by experience of people with personality disorders delivering training | 8 Experts by Experience | Age ranges from 25-65 years old | 8 Female | Ethnicity were all White | Borderline personality disorder | Narrative analytical framework and holistic analysis  Social constructionist approach | Themes found were termed chapters. Chapter 1 detailed the “screaming in a milk bottle” theme identifying acute experiences prior to becoming an expert by experience where their needs were not always visible or heard. They were also excluded from life changing decisions regarding treatment and diagnosis. Chapter 2 was a turning point where they understood their experiences because of problems with services and not themselves. Chapter 3 was about taking up the expert by experience role and how it turned experiences into something positive. Chapter 4 also discusses the emergence of the professional identity where they gain more power and value. Chapter 5 talked about impact on the self and others resulting in self-worth, and self-esteem. |
